# Comparative molecular profiling of pancreatic ductal adenocarcinoma of the head versus body and tail

**DOI:** 10.1038/s41698-024-00571-4

**Published:** 2024-04-06

**Authors:** Maen Abdelrahim, Abdullah Esmail, Anup Kasi, Nestor F. Esnaola, Joanne Xiu, Yasmine Baca, Benjamin A. Weinberg

**Affiliations:** 1 Section of GI Oncology, Houston Methodist Neal Cancer Center and Cockrell Center for Advanced Therapeutics, Houston Methodist Hospital, Houston, TX USA; 2 University of Kansas Medical Center, Kansas City, KS USA; 3 Department of Surgery, Houston Methodist Hospital, Houston, TX USA; 4 Caris Life Sciences, Phoenix, AZ USA; 5 Ruesch Center for the Cure of Gastrointestinal Cancers, Lombardi Comprehensive Cancer, Georgetown University Medical Center, Washington, DC USA

**Keywords:** Pancreatic cancer, Surgical oncology

## Abstract

Pancreatic ductal adenocarcinoma (PDAC) of the head (H) and body/tail (B/T) differ in embryonic origin, cell composition, blood supply, lymphatic and venous drainage, and innervation. We aimed to compare the molecular and tumor immune microenvironment (TIME) profiles of PDAC of the H vs. B/T. A total of 3499 PDAC samples were analyzed via next-generation sequencing (NGS) of RNA (whole transcriptome, NovaSeq), DNA (NextSeq, 592 genes or NovaSeq, whole exome sequencing), and immunohistochemistry (Caris Life Sciences, Phoenix, AZ). Significance was determined as *p* values adjusted for multiple corrections (*q*) of <0.05. Anatomic subsites of PDAC tumors were grouped by primary tumor sites into H (*N* = 2058) or B/T (*N* = 1384). There were significantly more metastatic tumors profiled from B/T vs. H (57% vs. 44%, *p* < 0.001). *KRAS* mutations (93.8% vs. 90.2%), genomic loss of heterozygosity (12.7% vs. 9.1%), and several copy number alterations (*FGF3*, *FGF4*, *FGF19*, *CCND1*, *ZNF703*, *FLT4*, *MUTYH*, *TNFRS14*) trended higher in B/T when compared to H (*p* < 0.05 but *q* > 0.05). Expression analysis of immuno-oncology (IO)-related genes showed significantly higher expression of *CTLA4* and *PDCD1* in H (*q* < 0.05, fold change 1.2 and 1.3) and *IDO1* and *PDCD1LG2* expression trended higher in B/T (*p* < 0.05, fold change 0.95). To our knowledge, this is one of the largest cohorts of PDAC tumors subjected to broad molecular profiling. Differences in IO-related gene expression and TIME cell distribution suggest that response to IO therapies may differ in PDAC arising from H vs. B/T. Subtle differences in the genomic profiles of H vs. B/T tumors were observed.

## Introduction

Pancreatic ductal adenocarcinoma (PDAC) is an aggressive cancer with a dramatically increasing incidence over the last decade, to the point that it currently represents the seventh greatest cause of cancer mortality in males and females globally^1,2^. This startling rise in rates of occurrence and mortality reflect the societal increases in obesity and diabetes and are, unfortunately, proving to be relatively stable, comparative to the declining trends of other cancers^3,4^. In light of this, and the mortality rate of PDAC already the highest among reported malignancies, statistical trends show that by 2030 it is expected to be the third greatest cause of cancer-related deaths worldwide, and the second leading cause of cancer-related mortality in the US^5,6^. Clinically, the absence of any diagnosable symptoms in the early stages of disease onset as well as the lack of any, standardly dependable, efficient screening or early detection tools, leaves the majority of PDAC patients being diagnosed in advanced unresectable stages with poor outcomes^7,8^. Additionally, despite all the resources and medical developments directed at PDAC, the 5-year survival rate has not advanced beyond 10% for the last 50 years^2,9^.

The standard curative measure for PDAC patients is resection, though transplantation and pancreatectomy are clinical options as well^10,11^. Even understanding that the majority of PDAC patients will present with late or advanced stages, those that are able to qualify for curative treatment contend heavily with high recurrence rates. As demonstrated in several clinical studies^12–14^, regardless to the inclusion of adjuvant chemotherapy, recurrence has still been reported as high as 91.1%^15^. Though the primary proportion of PDAC patients will be in stages past the point of curative intervention, the studies conducted in which relapse is observed can be utilized to better assess PDAC prognosis, which may aid in its management for better prospective outcomes. Among recent research, well-known prognostic variables for PDAC include tumor size and histological features are being utilized to create better comprehensive management regimens^16,17^. In addition to age, gender, pathological staging, serum cancer antigen 19-9 (CA19-9) level and oncogene mutations, such as KRAS, TP53, CDKN2A, and SMAD4, have been suggested as important prognostic variables for PDAC have also been administered in research to further assess disease progression, aggression, and survival outcomes^18–20^.

The pancreas is also separated into various anatomic regions, the uncinate process, the head (H), the body, and the tail. Given these anatomical variations, a protracted discussion has been launched to determine if the site of PDAC may influence the development of the tumor^21,22^. Numerous studies have shown significant variations in the prognosis of pancreatic tumors localized in the H versus those found in the body and tail (B/T)^23–26^. Tumors of the H and uncinate process are often accompanied by jaundice and are thus believed to manifest sooner in the disease’s progression. However, B/T pancreatic cancers often manifest with weight loss and discomfort, signs more consistent with advanced disease^27^. Although variations in prognosis have mostly been attributable to the late presentation of the B/T compared to PDAC of the H, earlier studies indicate that the tumor, lymph nodes, and metastasis TNM stage at presentation is not substantially different between the two tumor sites^28^.

The tumor immune microenvironment (TIME) profiles of PDAC are what have been found to be differently dysregulated depending on the tumor site. It appears that B/T tumors are highly proliferative in similar receptor expressions, compared to PDAC of the H, which has variant expressions^29^. In addition, B/T tumors had been shown to be more aggressive with a higher invasion and desmoplasia and a relatively poorer response to tumor immunotherapy^30^. Further analysis revealed a higher density of calcium-binding protein S100A2 mRNA transcripts in B/T tumors compared to a higher B cell signaling in PDAC of the H, which has been linked with favorable outcomes^29,31^. Our study aims to compare the molecular and TIME profiles of PDAC of the H vs. B/T, to determine a clinically notable difference that would dictate separate treatments modalities for primary tumor from these two separate origins.

## Results

### Cohort features

**Fig. 1:**
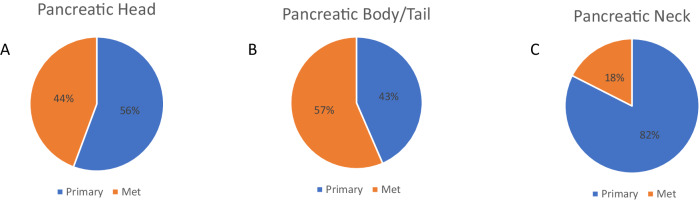
Primary/metastatic distribution in pancreatic adenocarcinoma cohorts. Head (**A**), body/tail (**B**), and neck (**C**).

We included a total of 3499 PDAC in the analysis. These cases were grouped anatomically by primary tumor sites into H (*N* = 2058) or B/T (*N* = 1384). Neck (*N* = 57) tumors were reported as a separate pancreatic anatomical subtype and not included in the comparative analysis. The median age for H tumors was (67) years old compared to (68) in B/T. There were significantly more metastatic tumors profiled from B/T compared to H (57% vs. 44%, *p* < 0.001) (Fig. 1). Table 1 summarizes the demographics based on the anatomical subdivisions of PDAC.

**Table 1:** Demographics of trial cohort, including tumor positioning, sex, and median age

	Total *N* (%)	Primary (%)	Metastatic (%)	Unclear (%)	Male (%)	Female (%)	Median age
Head	2058 (59)	1145 (64%)	912 (53%)	1 (100%)	1085 (58%)	973 (59%)	67
Body/Tail	1384 (39)	602 (33%)	782 (46%)	0 (0%)	746 (40%)	638 (39%)	68
Neck	57 (2)	47 (3%)	10 (1%)	0 (0%)	27 (2%)	30 (2%)	69
Total *N*	3499	1794	1704	1	1858	1641	

### Mutations features

**Fig. 2:**
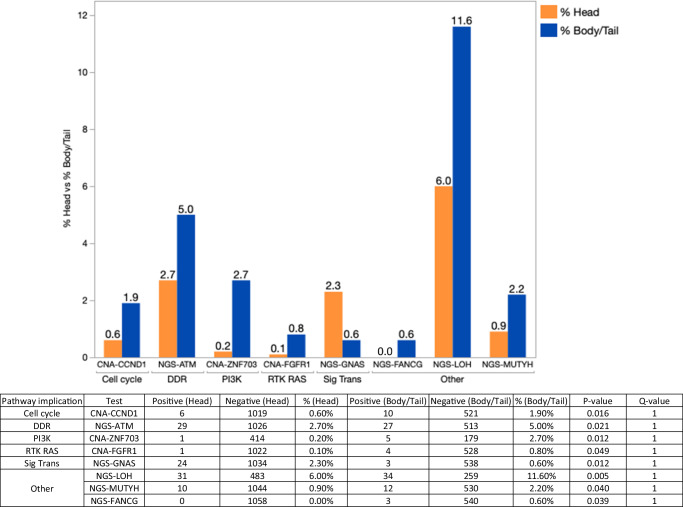
Trending alterations in H vs. B/T all *p* < 0.05 (*q* not significant). H head, B/T body/tail.

In our analysis, molecular alterations varied among B/T tumors and H tumors. For instance, KRAS mutations trended more prevalent in B/T vs. H tumors (93.8% vs. 90.2%). Other trends included genomic loss of heterozygosity (by whole exome sequencing) (12.7% vs. 9.1%), and several copy number alterations (FGF3 (2.2% vs. 1.1%), FGF4 (1.5% vs. 0.6%), FGF19 (1.7% vs. 0.9%), CCND1 (2.0% vs. 0.8%), ZNF703 (2.8% vs. 0.8%), FLT4 (1.1% vs. 0.4%), MUTYH (2.4% vs. 1.0%) and TNFRS14 (1.0% vs. 0.4%)) (all *p* < 0.05 but *q* > 0.05). In contrast, GNAS mutations (2.2% vs. 0.7%) trended higher in H vs. B/T (Fig. 2).

### Immuno-oncology features

**Fig. 3:**
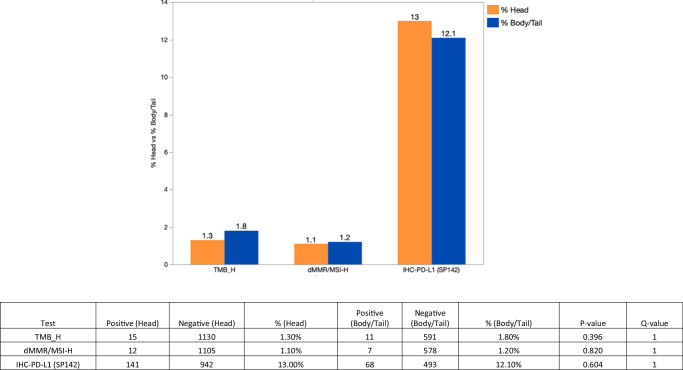
IO markers in H vs. B/T (no statistically significant difference observed). H head, B/T body/tail.

**Fig. 4:**
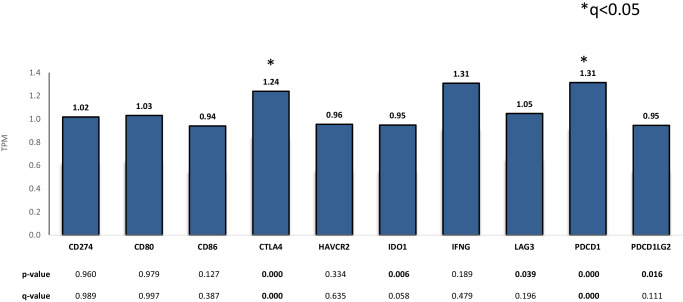
Expression analysis (fold change) of IO-related genes in H vs. B/T. H head, B/T body/tail.

Immuno-oncology (IO) markers, including TMB, PD-L1, and MSI-H showed no significant difference between B/T vs. H (Fig. 3), however, expression analysis of IO-related genes showed significantly higher expression of CTLA4 and PDCD1 in H (*q* < 0.05, fold change 1.2 and 1.3), compared to a higher expression of IDO1 and PDCD1LG2 in B/T (*p* < 0.05, fold change 0.95) shown in Fig. 4.

### Tumor immune microenvironment (TIME) features

**Fig. 5:**
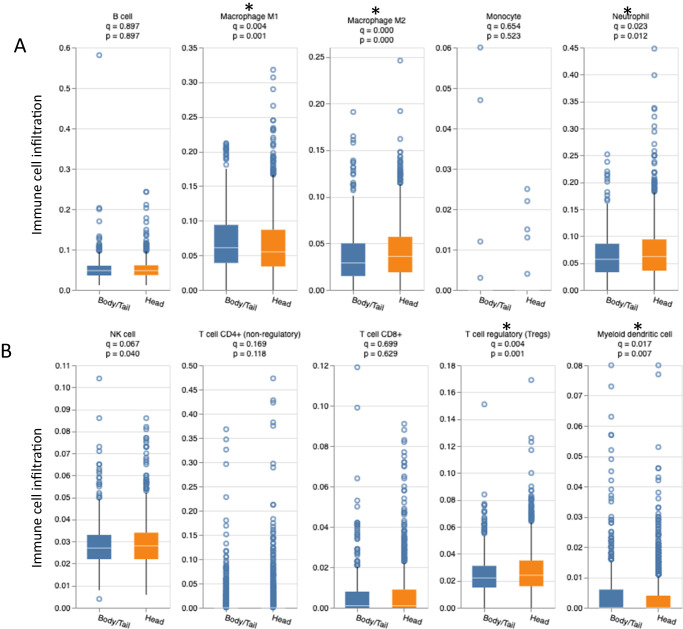
The tumor immune microenvironment for H vs. B/T. B cells, macrophages, monocytes, and neutrophils (**A**), NK cells, T cells, and myeloid dendritic cells (**B**). H head, B/T body/tail.

**Fig. 6:**
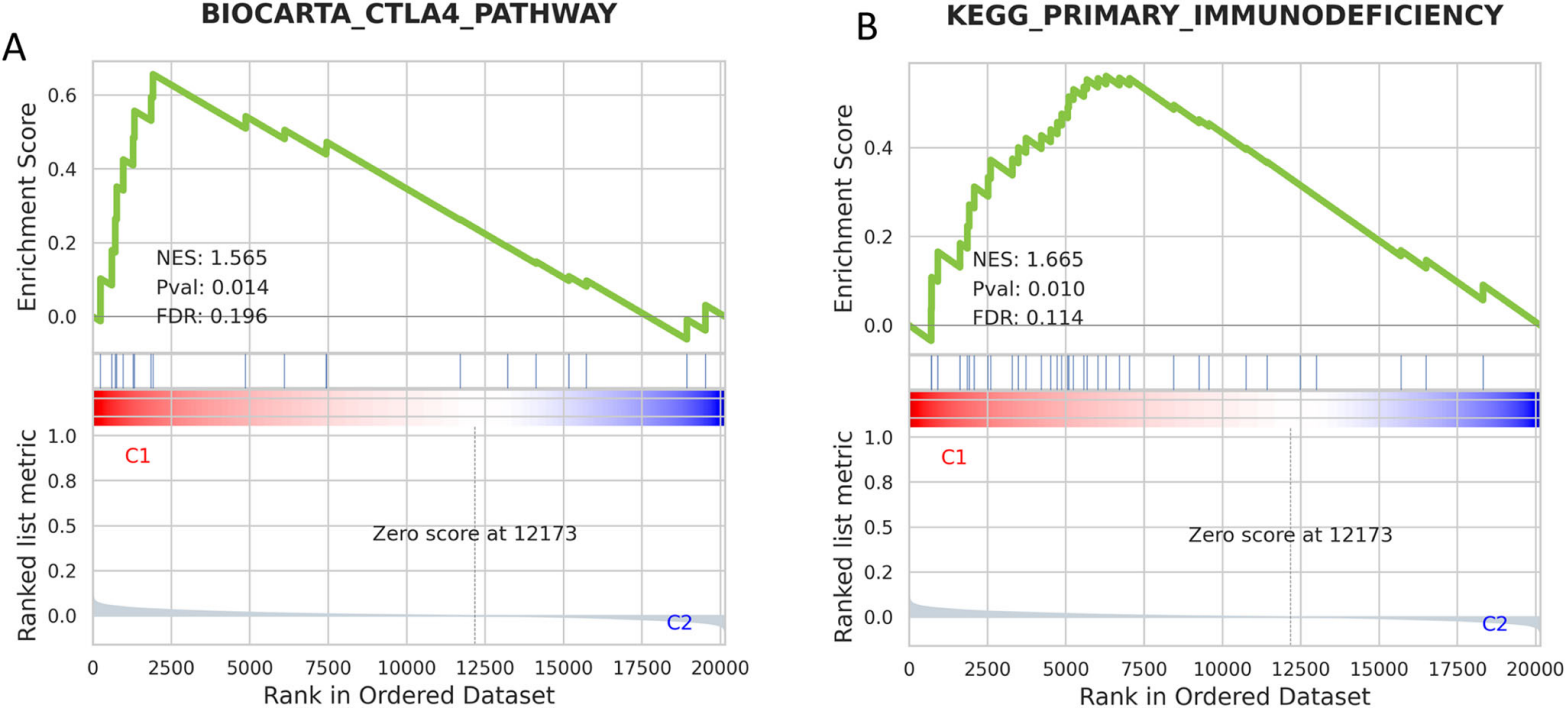
GSEA significant results, pathway enrichment in H compared to B/T. CTLA4 pathway (A) and primary immunodeficiency pathway (**B**). H head, B/T body/tail, CTLA-4 cytotoxic T-lymphocyte associated-4.

In order to evaluate the TIME, median cell abundance values were compared using QuantiSeq. Our analysis revealed that H tumors had increased immune infiltration of B cells (0.045 vs. 0.043), M2 macrophages (0.035 vs. 0.032), neutrophils (0.056 vs. 0.052), NK cells (0.027 vs. 0.026), CD8+ T cells (% > 0: 48.2% vs. 43.2%), while B/T had increased infiltration of M1 macrophages (0.035 vs. 0.032) (all *q* < 0.05) (Fig. 5). Pathway enrichment analysis using GESA showed that the CTLA4 pathway (Biocarta; normalized enrichment score (NES) 1.6, false discovery rate (FDR) 0.19) and primary immunodeficiency pathway (Kegg; NES 1.7, FDR 0.11) were significantly enriched in H compared to B/T (Fig. 6).

### Patients outcomes and prognostic features

**Fig. 7:**
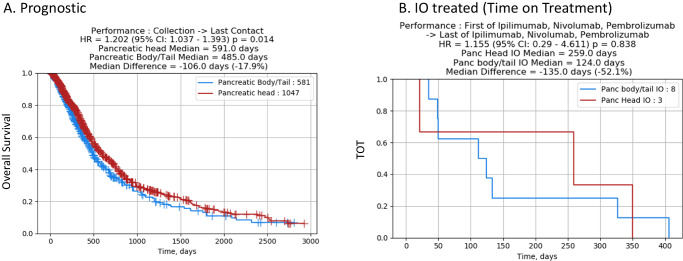
Outcomes in H vs. B/T, representing overall survival and time on treatment with immunotherapy, with time measured in days. Overall survival (**A**) and time on treatment with immunotherapy (**B**). H head, B/T body/tail, IO immuno-oncology, panc pancreatic.

Comparison of survival between H and B/T tumors showed that pancreatic H tumors had a prognostic advantage when compared to B/T (HR of 1.20; 95% CI 1.03–1.39, *p* = 0.014) (Fig. 7).

## Discussion

This retrospective study was designed to directly analyze the differential expressions in the molecular and TIME profiles of H and B/T tumors in patients with PDAC. Clinical evidence demonstrated that not only were there molecular and TIME differences in receptor expression between the H and the B/T, but patients with B/T were more likely to present somatic symptoms at later stages and were less likely to undergo resection^22,32^.

Several population-based studies^22,33–36^ conveyed variations in survival for both B/T and H with a mildly better observed survival in the early stages of those reported with B/T. This highlights a direct limitation to this study, the opposing clinical evidence, in which survival outcomes from B/T versus H volley between succession. A prime example of this is in the study by Ruess et al.^21^. where the postoperative survival of PDAC patients with B/T tumors appeared to be superior to that of PDAC patients with tumors positioned on the H. However, these findings are not universal, as various research revealed that survival rates are comparable for B/T and H tumors^22,36–38^. Furthermore, The SEER data (Surveillance, Epidemiology, and End Results Program of the National Cancer Institute) revealed poorer outcomes and increased mortality and morbidity in patients with a tumor located in the B/T than in PDAC of the H^39^. According to Winer et al.^34^ H patients who underwent early tumor resection were more likely to have higher-grade tumors with worse overall survival (OS) and higher numbers of positive lymph nodes. The improved survival of B/T population observed in that study is supported by a single-center study^40^ which examined survival in matched stage II B/T and H. Only one single-center study^41^ reported worse outcomes for B/T patients with non-statistically significant improved survival in stage I disease. Despite similar survival outcomes for both location types, our study observed subtle differences in gene expression and TIME cell distribution that may differ in H vs. B/T outcomes.

Consistent with previous reports^32,42^, the most common mutations in our study were KRAS, and were more prevalent in B/T vs. H tumors (93.8% vs. 90.2%). Though, a more recent study found that only TP53 mutations were significantly higher in B/T as well as the different clinical presentations. Moreover, the molecular profiles emphasized that the H and B/T are different tumors in addition to having differentiating OS, molecular profiles, and response to treatments^43^. However, our study showed genomic loss of heterozygosity and numerous alterations (FGF3, FGF4, FGF19, CCND1, ZNF703, FLT4, MUTYH, TNFRS14) that were higher in B/T when compared to H (*p* < 0.05 but *q* > 0.05). In contrast, GNAS mutations (2.2% vs. 0.7%) were higher in H vs. B/T. Though there was a past study by Wu et al.^44^ that demonstrated pathways in papillary mucinous neoplasm (IPMN) and GNAS mutation in identifying adenocarcinoma in pancreatic cysts there is literature differentiating predicating tumor mutations of the H vs. the B/T. Additionally, regarding the study of Sun et al., the total number of SMAD mutations was 12.12% rendering elevated SMAD mutations in H (15.5%) compared to B/T(5%) statistically insignificant because biopsy specimens or peripheral blood (66.7%) made up the majority of tissue samples and this may have lowered the detection of certain mutations.

In this study, expression analysis of IO-related genes showed significantly higher expression of CTLA-4 and PDCD1 in H (*q* < 0.05, fold change 1.2 and 1.3), compared to a higher expression of IDO1 and PDCD1LG2 in B/T (*p* < 0.05, fold change 0.95). These findings indicate that the response to IO treatments might be varied in PDAC originating from H vs. B/T. The results of our study are also consistent with the previous study by Sun et al. that reported a different treatment response that was explained by greater TP53 mutations in B/T, indicating that gemcitabine-based adjuvant therapy should be considered in treating B/T pancreatic cancer. TP53 mutation is commonly seen in pancreatic squamous cell cancer^31^. TP53 positively predicted sensitivity to gemcitabine-based adjuvant therapy in survival and mutational analysis from the CONKOO-001 study^42^. Taking the results of this study into account, the broad-spectrum treatment of patients with PDAC may be re-evaluated to account for the patient’s tumor origin. Transitioning to the focus of molecular and TIME-based treatment may be used to further promote better outcomes for both B/T and H PDAC patients by separating their regimens to account for receptor expression.

To our knowledge, this study is the largest cohort of PDAC tumors subjected to broad molecular profiling. Our study demonstrated differences in IO-related gene expression and TIME cell distribution suggesting that response to immunotherapies may differ in pancreatic cancer arising from the pancreatic H versus the pancreatic B/T^45^. Moreover, subtle differences in the genomic profiles of pancreatic H versus B/T tumors were also observed in this study, might play a crucial role in supporting overall response regarding the interventions of coupling chemo/immunotherapy, specifically PD-1 and CTLA-4 inhibitors as well as improve the OS of PDAC. The biological criteria that established pancreatic H adenocarcinoma as a specific patient population might lead to improvements in the overall response to study immunotherapies interventions as well as giving us more accurate data on the pancreatic H adenocarcinoma responses to immunotherapies. Given the potential impact of tumor location on pancreatic cancer prognosis, more studies to determine the broader molecular profiling aimed to directly compare the TIME profiles of pancreatic cancer H versus B/T microenvironments are essential for establishing improved treatment regimens. One of this study’s limitations is that it is a retrospective analysis. Additionally, this study hasn’t reported the treatments data among its sample size that lacks the opportunity of more transparency to analyze and report the real microenvironment implications that could be affected by IO. More clinical trials in a prospective setting need to be performed in a prospective manner to potentially guide clinical practice. In conclusion and based on our knowledge, this is one of the largest cohorts of PDAC tumors that has undergone extensive molecular profiling. Differences in IO-related gene expression and TIME cell distribution imply that PDAC resulting from the H vs. B/T may respond differently to IO therapies. The genomic profiles of H vs. B/T pancreatic tumors showed slight variations as well.

## Methods

### Patient cohort

PDAC tumors were submitted to Caris Life Sciences (Phoenix, AZ). Tumors were then categorized according to primary tumor sites; H of the pancreas or B/T of the pancreas for analysis. This study was conducted in accordance with the guidelines of the Declaration of Helsinki, the Belmont report, and the U.S. Common rule. In keeping with 45 CFR 46.101(b)^4^, this study was performed utilizing retrospective, de-identified clinical data. Therefore, this study is considered IRB exempt and no patient consent was necessary from the subject.

### Next-generation sequencing (NGS)

Next-generation sequencing (NGS) (Illumina Next Seq, 592 genes) was performed on genomic DNA isolated from Formalin-Fixed Paraffin-Embedded FFPE samples. All variants were detected with >99% confidence based on allele frequency and amplicon coverage, with an average sequencing depth of coverage of >500 and an analytic sensitivity of 5%. Prior to molecular testing, tumor enrichment was achieved by harvesting targeted tissue using manual microdissection techniques. The genetic variants identified were interpreted by board-certified molecular geneticists and categorized as “pathogenic”, “presumed pathogenic”, “variant of unknown significance”, “presumed benign”, or “benign”, according to the American College of Medical Genetics and Genomics (ACMG) standards. When assessing mutation frequencies of individual genes, “pathogenic” and “presumed pathogenic” were counted as mutations while “benign”, “presumed benign” variants, and “variants of unknown significance” were excluded.

### TMB

TMB was measured by counting all non-synonymous missense, nonsense, in frame insertion/deletion, and frameshift mutations found per tumor that had not been previously described as germline alterations in dbSNP151, Genome Aggregation Database (gnomAD) databases or benign variants identified by Caris’s geneticists. A cutoff point of ≥10 mutations per MB was used based on the KEYNOTE-158 pembrolizumab trial^46^, which showed that patients with a TMB of ≥10 mt/MB across several tumor types had higher response rates than patients with a TMB of <10 mt/MB. Caris Life Sciences is a participant in the Friends of Cancer Research TMB Harmonization Project^47^.

### mRNA expression (WTS)

Tumors underwent RNA sequencing using full formalin-fixed paraffin-embedded (FFPE) specimens that were reviewed by a board-certified pathologist to measure percent tumor content and tumor size; a minimum of 20% of tumor content in the area for microdissection was required to enable enrichment and extraction of tumor-specific RNA. A Qiagen RNA FFPE tissue extraction kit was used for extraction, and the RNA quality and quantity were determined using the Agilent TapeStation. Biotinylated RNA baits were hybridized to synthesized and purified cDNA targets and the bait-target complexes were amplified in a post-capture PCR reaction. The Illumina NovaSeq 6500 was used to sequence the whole transcriptome from patients to an average of 60 M reads. Raw data was demultiplexed by Illumina Dragen Bio-IT accelerator, trimmed, counted, PCR-duplicates removed, and aligned to the human reference genome hg19 by STAR aligner. For transcription counting, transcripts per million molecules were generated using the Salmon expression pipeline. Human All Exon V7 bait panel (Agilent Technologies, Santa Clara, CA) was prepared. Immune cell fraction was calculated by QuantiSeq using this transcriptomic data^48^. Additionally, this mRNA data was used as input for pathway gene enrichment analyses using Gene Set Enrichment Analysis^49^.

### Data and statistical analysis

The prevalence of molecular alterations among cohorts were analyzed using Chi-square or Fisher Exact tests. Expression distribution among cohorts were analyzed using non-parametric Kruskal–Wallis’s testing. Similarly, tumor microenvironment cell fractions were analyzed as described previously. A value of <0.05 was considered a trending difference; *p* values were further corrected for multiple comparisons using the Benjamini–Hochberg method to avoid type I error and an adjusted *p* value (i.e., *q* value) of <0.05 was considered a significant difference.

### Outcomes analysis

Real-world overall survival (rwOS) information was obtained from insurance claims data and calculated from the time of tissue collection to the last contact or treatment time to last treatment time (TOT). Kaplan–Meier estimates were calculated for molecularly defined patient cohorts. Significance was determined as *p* values < 0.05.

### Reporting summary

Further information on research design is available in the Nature Research Reporting Summary linked to this article.

### Supplementary information

Supplemental Material 3

REPORTING SUMMARY

## Data Availability

The datasets generated during and/or analyzed during the current study are available from the corresponding author on reasonable request. The de-identified sequencing data cannot be publicly shared due to the data usage agreement between the facilities of the study team. Qualified researchers can apply for access to these summarized data by contacting J.X. and signing a data usage agreement. The processed NGS data are available at: (summary table of primary tumors to match figures attached). Other questions regarding the data of this study are welcomed on request to the corresponding author, B.A.W.
