# Supplementary material for: Comparative molecular profiling of pancreatic ductal adenocarcinoma of the head versus body and tail
Source: NPJ Precis Oncol. 2024 Apr 6;8:85. doi: 10.1038/s41698-024-00571-4 (PMC10998911; doi:10.1038/s41698-024-00571-4)
Supplement: Supplementary file 1 — Supplemental Material 3 [file 41698_2024_571_MOESM1_ESM.pptx]

## Slide 1
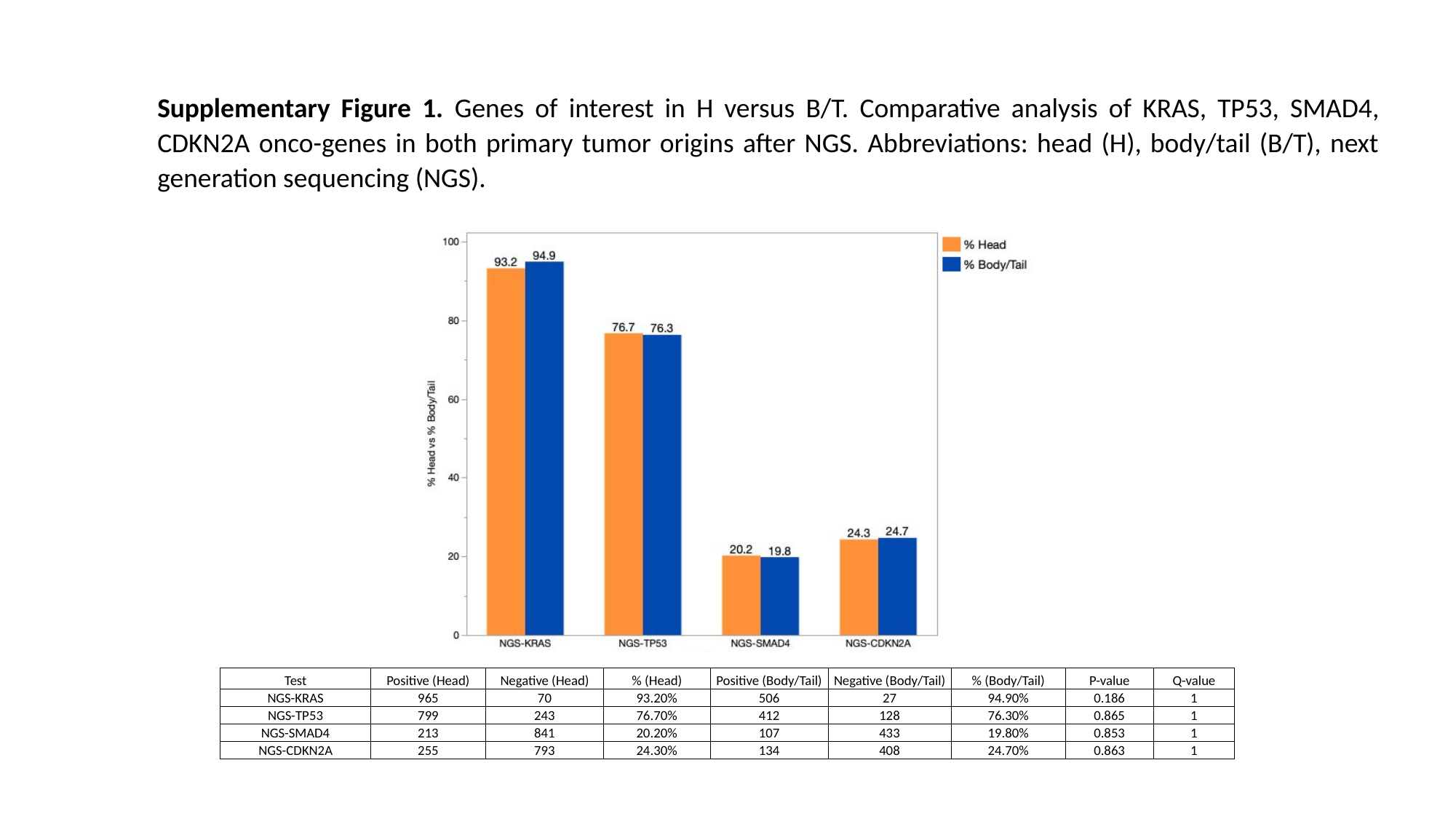

Supplementary Figure 1. Genes of interest in H versus B/T. Comparative analysis of KRAS, TP53, SMAD4, CDKN2A onco-genes in both primary tumor origins after NGS. Abbreviations: head (H), body/tail (B/T), next generation sequencing (NGS).
| Test | Positive (Head) | Negative (Head) | % (Head) | Positive (Body/Tail) | Negative (Body/Tail) | % (Body/Tail) | P-value | Q-value |
| --- | --- | --- | --- | --- | --- | --- | --- | --- |
| NGS-KRAS | 965 | 70 | 93.20% | 506 | 27 | 94.90% | 0.186 | 1 |
| NGS-TP53 | 799 | 243 | 76.70% | 412 | 128 | 76.30% | 0.865 | 1 |
| NGS-SMAD4 | 213 | 841 | 20.20% | 107 | 433 | 19.80% | 0.853 | 1 |
| NGS-CDKN2A | 255 | 793 | 24.30% | 134 | 408 | 24.70% | 0.863 | 1 |

## Slide 2
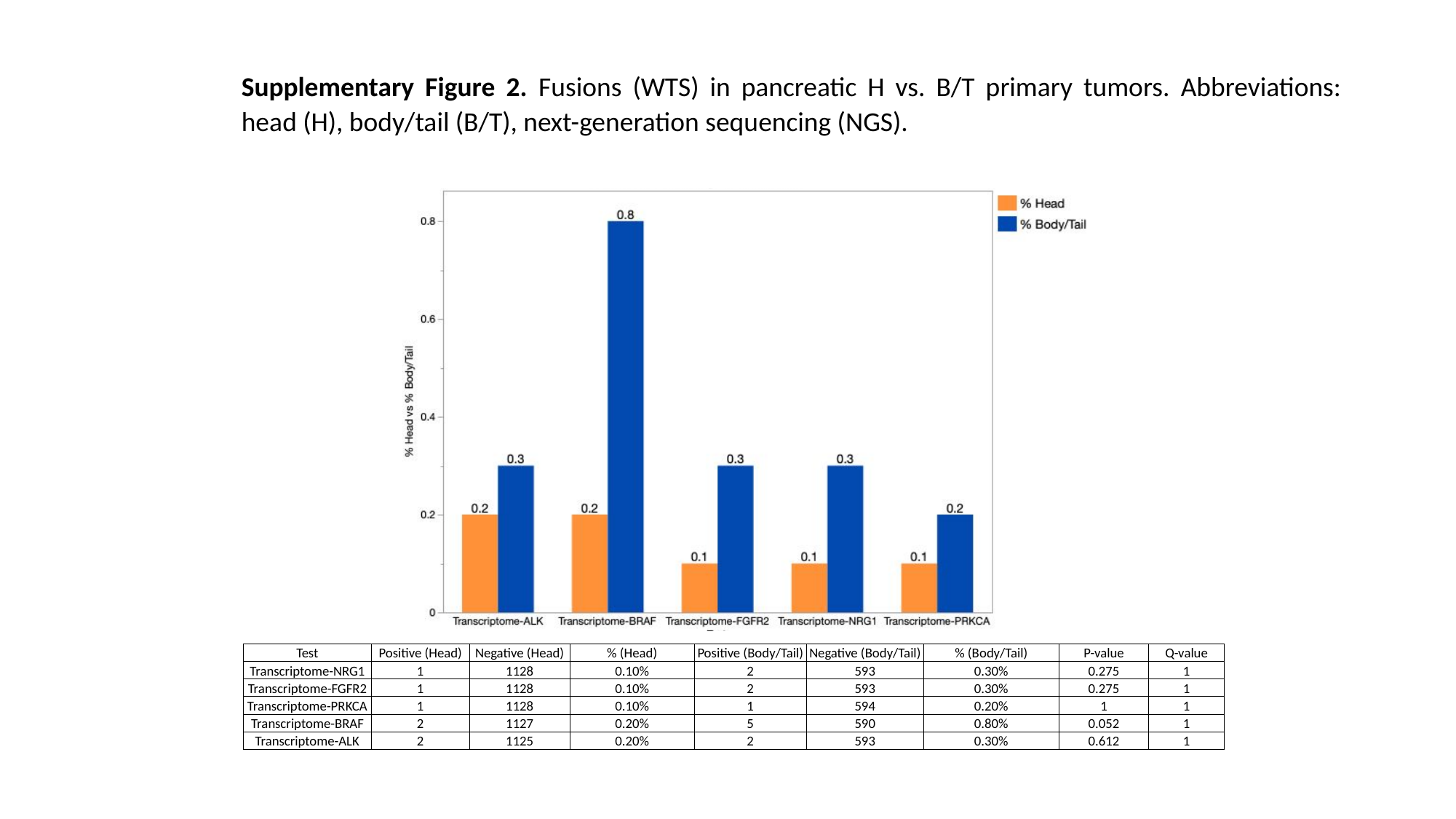

Supplementary Figure 2. Fusions (WTS) in pancreatic H vs. B/T primary tumors. Abbreviations: head (H), body/tail (B/T), next-generation sequencing (NGS).
| Test | Positive (Head) | Negative (Head) | % (Head) | Positive (Body/Tail) | Negative (Body/Tail) | % (Body/Tail) | P-value | Q-value |
| --- | --- | --- | --- | --- | --- | --- | --- | --- |
| Transcriptome-NRG1 | 1 | 1128 | 0.10% | 2 | 593 | 0.30% | 0.275 | 1 |
| Transcriptome-FGFR2 | 1 | 1128 | 0.10% | 2 | 593 | 0.30% | 0.275 | 1 |
| Transcriptome-PRKCA | 1 | 1128 | 0.10% | 1 | 594 | 0.20% | 1 | 1 |
| Transcriptome-BRAF | 2 | 1127 | 0.20% | 5 | 590 | 0.80% | 0.052 | 1 |
| Transcriptome-ALK | 2 | 1125 | 0.20% | 2 | 593 | 0.30% | 0.612 | 1 |

## Slide 3
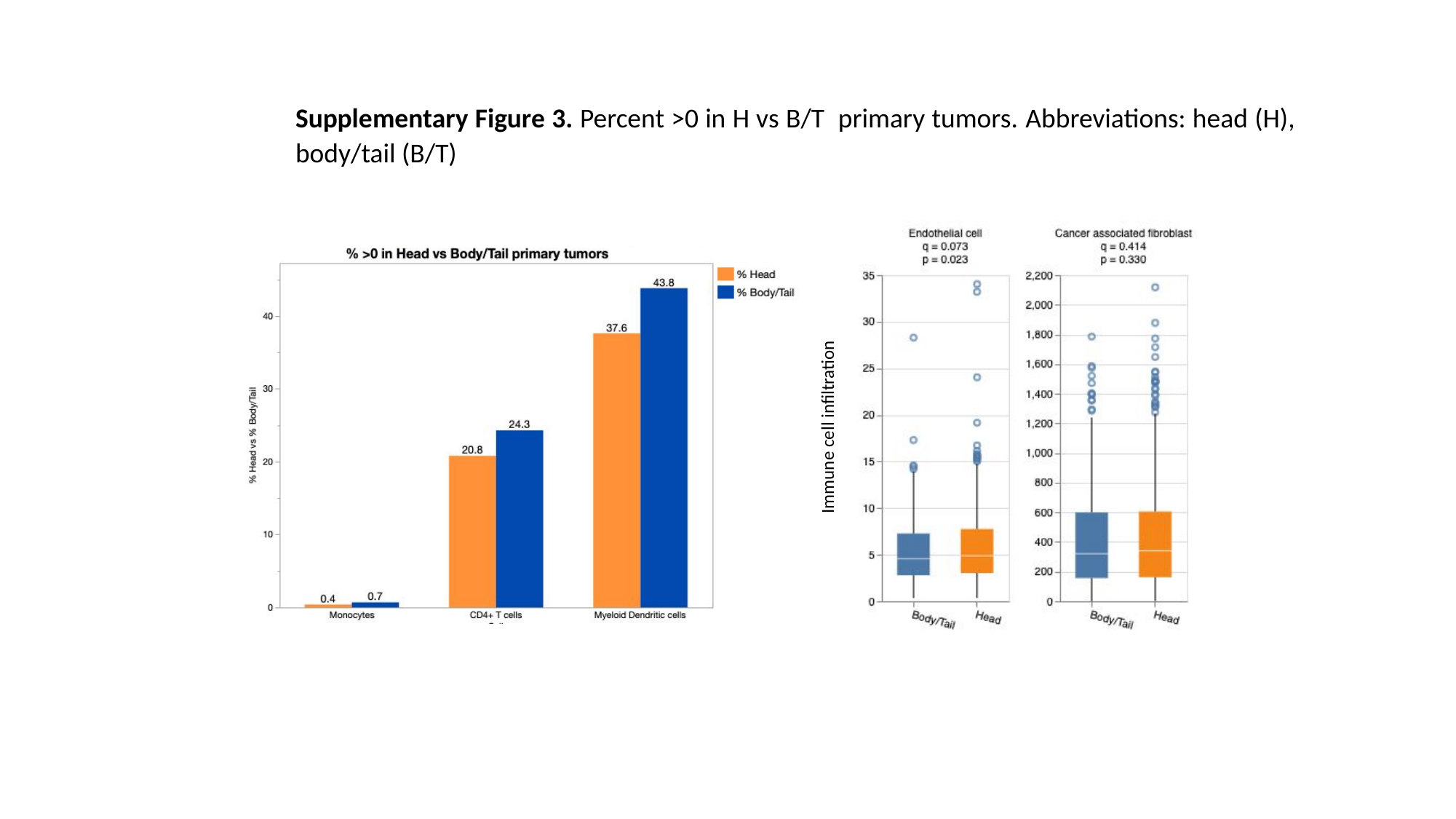

Supplementary Figure 3. Percent >0 in H vs B/T primary tumors. Abbreviations: head (H), body/tail (B/T)
Immune cell infiltration

## Slide 4
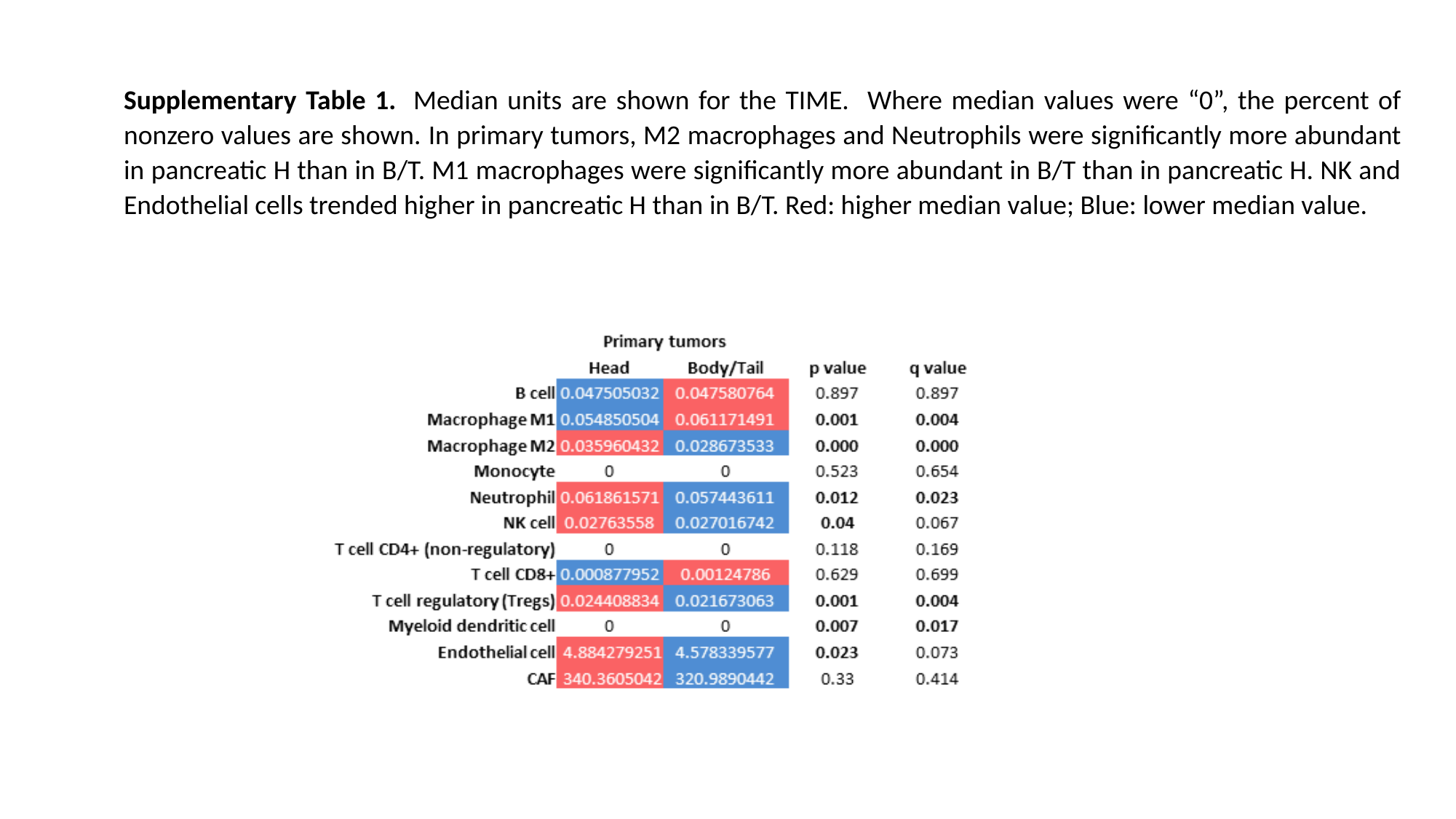

Supplementary Table 1. Median units are shown for the TIME. Where median values were “0”, the percent of nonzero values are shown. In primary tumors, M2 macrophages and Neutrophils were significantly more abundant in pancreatic H than in B/T. M1 macrophages were significantly more abundant in B/T than in pancreatic H. NK and Endothelial cells trended higher in pancreatic H than in B/T. Red: higher median value; Blue: lower median value.
