# Supplementary material for: Comparative molecular profiling of pancreatic ductal adenocarcinoma of the head versus body and tail
Source: NPJ Precis Oncol. 2024 Apr 6;8:85. doi: 10.1038/s41698-024-00571-4 (PMC10998911; doi:10.1038/s41698-024-00571-4)
Supplement: Supplementary file 2 — REPORTING SUMMARY [file 41698_2024_571_MOESM2_ESM.pdf]

nature portfolio

Corresponding author(s): Benjamin Weinberg,MD

Last updated by author(s): 12/20/2023

Reporting Summary

Nature Portfolio wishes to improve the reproducibility of the work that we publish. This form provides structure for consistency and transparency in reporting. For further information on Nature Portfolio policies, see our Editorial Policies and the Editorial Policy Checklist.

Statistics

For all statistical analyses, confirm that the following items are present in the figure legend, table legend, main text, or Methods section.

n/a	Confirmed	
☐	☒	The exact sample size (*n*) for each experimental group/condition, given as a discrete number and unit of measurement
☐	☒	A statement on whether measurements were taken from distinct samples or whether the same sample was measured repeatedly
☐	☒	The statistical test(s) used AND whether they are one- or two-sided<br>*Only common tests should be described solely by name; describe more complex techniques in the Methods section.*
☐	☒	A description of all covariates tested
☐	☒	A description of any assumptions or corrections, such as tests of normality and adjustment for multiple comparisons
☐	☒	A full description of the statistical parameters including central tendency (e.g. means) or other basic estimates (e.g. regression coefficient) AND variation (e.g. standard deviation) or associated estimates of uncertainty (e.g. confidence intervals)
☐	☒	For null hypothesis testing, the test statistic (e.g. *F*, *t*, *r*) with confidence intervals, effect sizes, degrees of freedom and *P* value noted<br>*Give P values as exact values whenever suitable.*
☐	☒	For Bayesian analysis, information on the choice of priors and Markov chain Monte Carlo settings
☐	☒	For hierarchical and complex designs, identification of the appropriate level for tests and full reporting of outcomes
☐	☒	Estimates of effect sizes (e.g. Cohen's *d*, Pearson's *r*), indicating how they were calculated

Our web collection on statistics for biologists contains articles on many of the points above.

Software and code

Policy information about availability of computer code

Data collection	PDAC tumors were submitted to Caris Life Sciences (Phoenix, AZ). Tumors were then categorized according to primary tumor sites; H of the pancreas or B/T of the pancreas for analysis. This study was conducted in accordance with the guidelines of the Declaration of Helsinki, the Belmont report, and the U.S. Common rule. In keeping with 45 CFR 46.101(b)(4), this study was performed utilizing retrospective, de-identified clinical data. Therefore, this study is considered IRB exempt and no patient consent was necessary from the subject.
Data analysis	The prevalence of molecular alterations among cohorts were analyzed using Chi-square or Fisher Exact tests. Expression distribution among cohorts were analyzed using non-parametric Kruskal-Wallis's testing. Similarly, tumor microenvironment cell fractions were analyzed as described previously. A value of <0.05 was considered a trending difference; p values were further corrected for multiple comparisons using the Benjamini-Hochberg method to avoid type I error and an adjusted p-value (i.e., q value) of <0.05 was considered a significant difference Real-world overall survival (rwOS) information was obtained from insurance claims data and calculated from the time of tissue collection to the last contact or treatment time to last treatment time (TOT). Kaplan-Meier estimates were calculated for molecularly defined patient cohorts. Significance was determined as p values <0.05.

For manuscripts utilizing custom algorithms or software that are central to the research but not yet described in published literature, software must be made available to editors and reviewers. We strongly encourage code deposition in a community repository (e.g. GitHub). See the Nature Portfolio guidelines for submitting code & software for further information.

Data

Policy information about availability of data

All manuscripts must include a data availability statement. This statement should provide the following information, where applicable:

- Accession codes, unique identifiers, or web links for publicly available datasets
- A description of any restrictions on data availability
- For clinical datasets or third party data, please ensure that the statement adheres to our policy

Provide your data availability statement here.

Research involving human participants, their data, or biological material

Policy information about studies with human participants or human data. See also policy information about sex, gender (identity/presentation), and sexual orientation and race, ethnicity and racism.

Reporting on sex and gender	Checked
Reporting on race, ethnicity, or other socially relevant groupings	confirmed
Population characteristics	applied
Recruitment	confirmed
Ethics oversight	Checked

Note that full information on the approval of the study protocol must also be provided in the manuscript.

Field-specific reporting

Please select the one below that is the best fit for your research. If you are not sure, read the appropriate sections before making your selection.

☒ Life sciences ☐ Behavioural & social sciences ☐ Ecological, evolutionary & environmental sciences

For a reference copy of the document with all sections, see nature.com/documents/nr-reporting-summary-flat.pdf

Life sciences study design

All studies must disclose on these points even when the disclosure is negative.

Sample size	3499
Data exclusions	checked
Replication	Checked
Randomization	NA
Blinding	NA

Reporting for specific materials, systems and methods

We require information from authors about some types of materials, experimental systems and methods used in many studies. Here, indicate whether each material, system or method listed is relevant to your study. If you are not sure if a list item applies to your research, read the appropriate section before selecting a response.

Materials & experimental systems

n/a	Involved in the study
☒	☐ Antibodies
☒	☐ Eukaryotic cell lines
☒	☐ Palaeontology and archaeology
☒	☐ Animals and other organisms
☒	☐ Clinical data
☒	☐ Dual use research of concern
☒	☐ Plants

Methods

n/a	Involved in the study
☒	☐ ChIP-seq
☒	☐ Flow cytometry
☒	☐ MRI-based neuroimaging

Plants

Seed stocks

NA

Novel plant genotypes

NA

Authentication

NA
